# Three new synonyms of the genus *Kamimuria* (Plecoptera, Perlidae)

**DOI:** 10.3897/BDJ.13.e153697

**Published:** 2025-05-23

**Authors:** Liang-Liang Zeng, Qing-Bo Huo, Yu-Zhou Du

**Affiliations:** 1 College of Plant Protection & Institute of Applied Entomology, Yangzhou University, Yangzhou 225009, China College of Plant Protection & Institute of Applied Entomology, Yangzhou University Yangzhou 225009 China; 2 Joint International Research Laboratory of Agriculture and Agri-Product Safety, the Ministry of Education, Yangzhou University, Yangzhou 225009, China Joint International Research Laboratory of Agriculture and Agri-Product Safety, the Ministry of Education, Yangzhou University Yangzhou 225009 China

**Keywords:** Plecoptera, Perlidae, *Kamimuria*, synonym, China

## Abstract

**Background:**

Currently, 11 species of *Kamimuria* have been reported in Guizhou Province, China. However, the original illustrations of *Kamimuriamagnimacula* Du, 2005 and *K.extremispina* Du, 2006, lack the necessary detail to accurately assess the spine patterns on the endophallus, which is a key diagnostic feature. To resolve this issue, a re-examination of the type materials, complemented by high-resolution colour photographs, is crucial to ensure precise identification and reliable documentation of these species.

**New information:**

Based on a detailed examination of the type materials of *Kamimuriamagnimacula* Du, 2005 and *K.extremispina* Du, 2006, we propose that *K.hunanensis* Li & Li, 2022 be considered a synonym of *K.magnimacula*, *K.circumspina* Li, Mo & Yang, 2019 and *K.dabieshana* Yan, Kong & Li, 2021 be regarded as synonyms of *K.extremispina*. Additionally, we have provided holotype photographs of *K.magnimacula* and *K.extremispina*, along with a distribution map for both species in this paper.

## Introduction

*Kamimuria* Klapálek, 1907 represents one of the most diverse genera within the subfamily Perlinae. Over the past two decades, taxonomic studies have revealed a significant increase in the number of described *Kamimuria* species (Du 1999, Yang and Li 2018, Du 2020, DeWalt et al. 2025). Guizhou Province in China is bordered by Sichuan Province and Chongqing Municipality to the north, Hunan Province to the east, Guangxi Zhuang Autonomous Region to the south and Yunnan Province to the west. To date, a total of 11 species of *Kamimuria* have been recorded in Guizhou (Li and Li 2022). However, the morphological characteristics of some species, particularly the original illustrations of the penis, are insufficiently detailed and have lacked comprehensive descriptions for an extended period, leading to difficulties in identification. Therefore, it is necessary to review the *Kamimuria* specimens collected from Guizhou and provide high-quality colour photographs to facilitate accurate identification and documentation.

**Figure 1.**
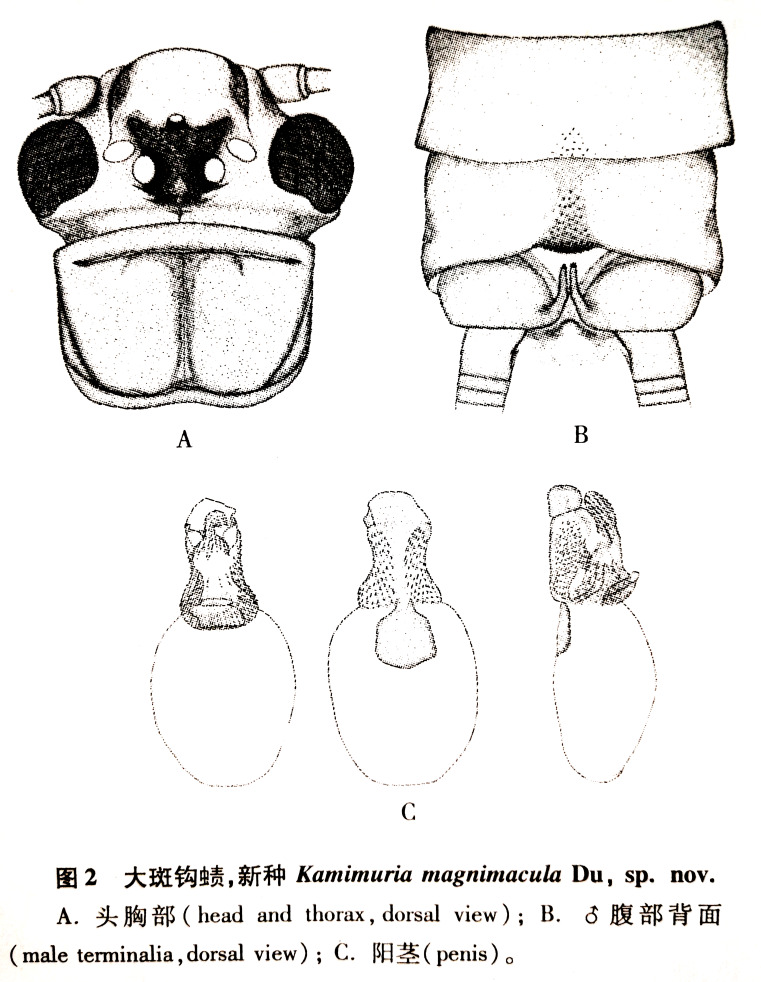
Original illustrations of *Kamimuriamagnimacula*, male. **A** head and pronotum, dorsal view; **B** abdominal terminalia, dorsal view; **C** everted penis (from Du and Wang (2005)).

**Figure 2.**
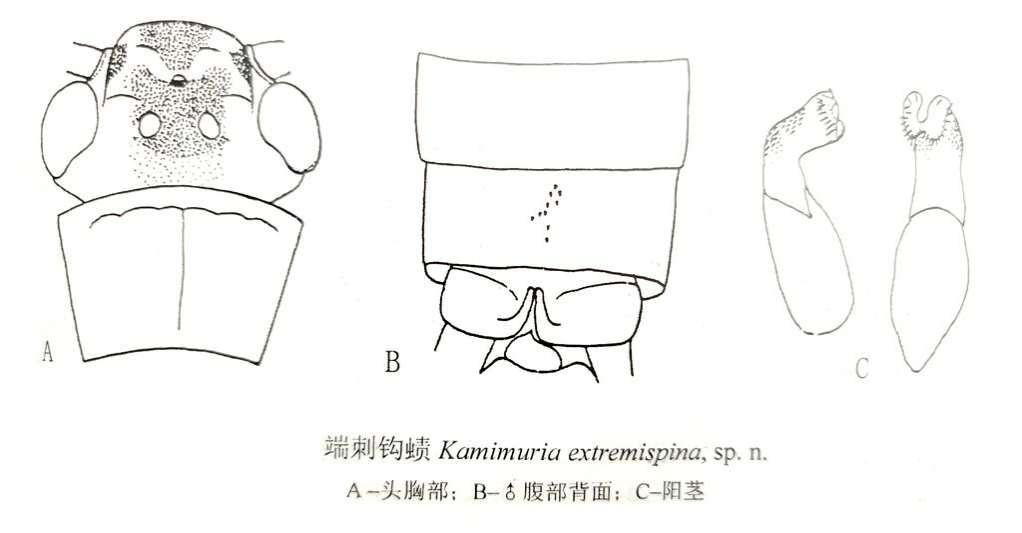
Original illustrations of *Kamimuriaextremispina*, male. **A** head and pronotum, dorsal view; **B** abdominal terminalia, dorsal view; **C** everted penis (from Du (2006)).

Recently, we conducted a detailed examination of specimens collected from Guizhou Province and identified specimens of *K.magnimacula* Du, 2005 (holotype from Guizhou) and *K.extremispina* Du, 2006 (paratype from Guizhou). However, based solely on the original illustrations of these two species, it was not possible to accurately determine the spine patterns on the endophallus (Figs 1, 2). To address this, we examined and photographed additional type material of these species for further analysis. Notably, we found that the male adult morphology of *K.hunanensis* Li & Li, 2022 (holotype from Hunan Province, China) is identical to that of *K.magnimacula*. Similarly, the male adult morphology of *K.circumspina* Li, Mo & Yang, 2019 (holotype from Guizhou Province, China) and *K.dabieshana* Yan, Kong & Li, 2021 (holotype from Hubei Province, China) is identical to that of *K.extremispina*. Based on further confirmation, we propose that *K.hunanensis* be considered a synonym of *K.magnimacula*, *K.circumspina* and *K.dabieshana* be regarded as synonyms of *K.extremispina*.

## Materials and methods

Specimens were collected by light trap. All materials were preserved in 75% ethanol and the penis were everted using the cold maceration technique of Zwick (1983). Photographs were taken with the KEYENCE VHX-5000 system and subsequently optimised in Adobe Photoshop CS6. All specimens were deposited in the Insect Collection of Yangzhou university (ICYZU), Jiangsu Province, China. Terminology followed Sivec and Stark (2008) and Zeng et al. (2024).

## Taxon treatments

### Kamimuria magnimacula

Du, 2005

9E788B0C-967F-5508-9BFA-10A22B831784

Kamimuria magnimacula Du, 2005 in Du & Wang, 2005: 53, 57. Stark & Sivec, 2013: 117. Yang & Li, 2018: 30. *Kamimurihunanensis* Li & Li, 2022:119. **syn. nov.**

#### Materials

- **Type status:** Holotype. **Occurrence:** recordedBy: Wang Zhijie; individualCount: 1; sex: male; lifeStage: adult; **Location:** country: China; stateProvince: Guizhou; county: Daozhen Gelao and Miao Autonomous; locality: Da Shahe National Nature Reserve; **Event:** year: 2004; month: 8; day: 22; **Record Level:** language: en
- **Type status:** Other material. **Occurrence:** recordedBy: Wang Zhijie; individualCount: 2; sex: male; lifeStage: adult; **Location:** country: China; stateProvince: Guizhou; county: Daozhen Gelao and Miao Autonomous; locality: Da Shahe National Nature Reserve; **Event:** year: 2004; month: 8; day: 22

#### Distribution

China (Guizhou, Hunan)

#### Taxon discussion

**Figure 3.**
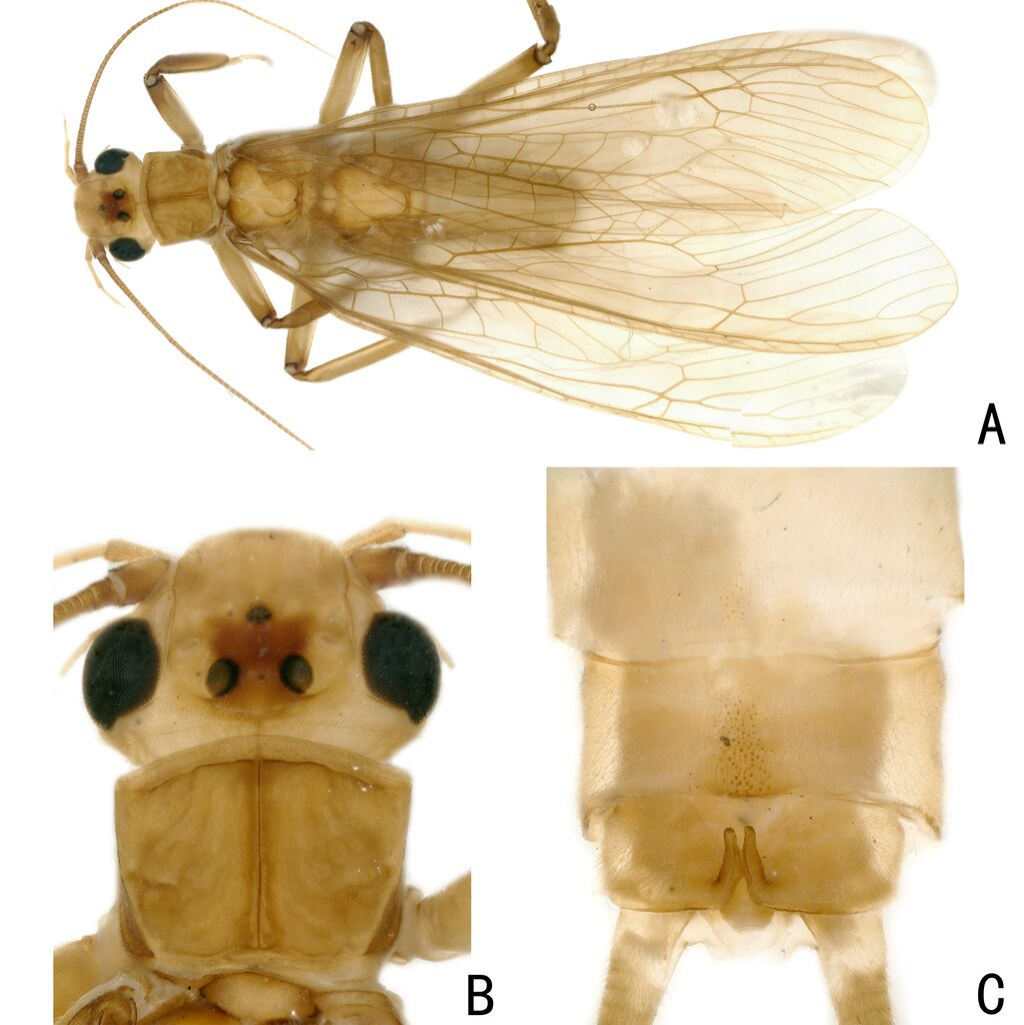
*Kamimuriamagnimacula*, male holotype. **A** dorsal habitus; **B** head and pronotum, dorsal view; **C** abdominal terminalia, dorsal view.

**Figure 4.**
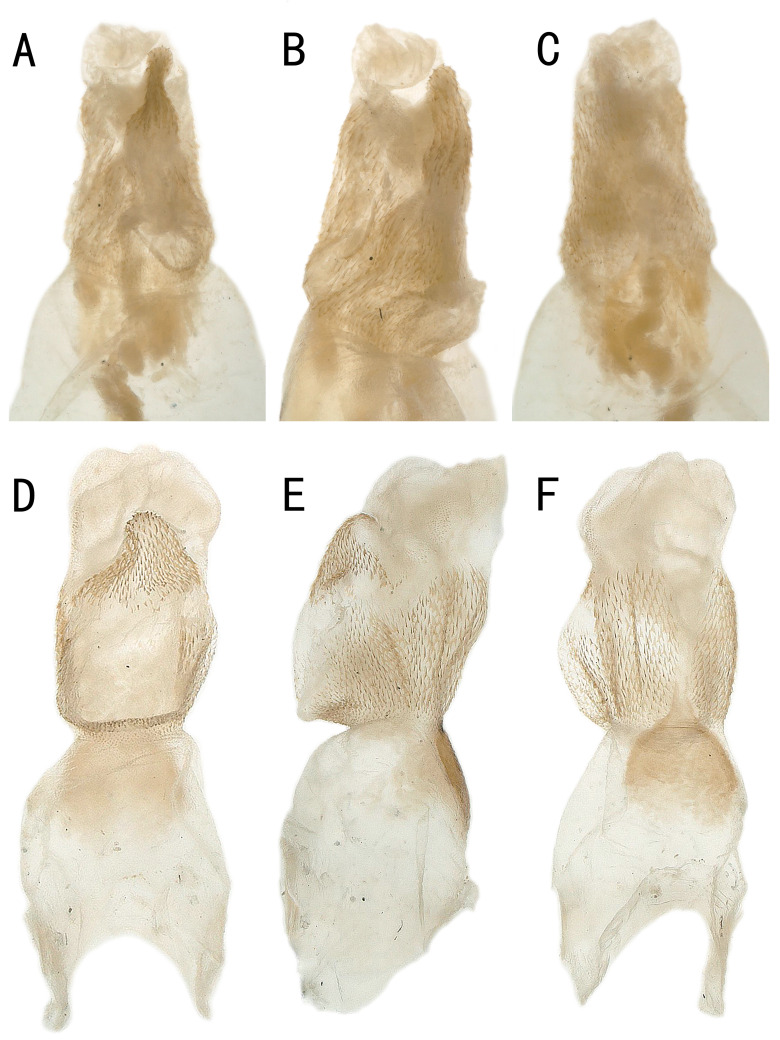
*Kamimuriamagnimacula*, holotype, everted penis. **A** dorsal view; **B** lateral view; **C** ventral view. *Kamimuriamagnimacula* shares the same data as holotype, **D** dorsal view; **E** lateral view; **F** ventral view.

It has been twenty years since the last report of *K.magnimacula* from Guizhou Province, currently, only hand-drawn illustrations of this species are available. To facilitate future identification efforts, we conducted a detailed examination of the type materials of *K.magnimacula*. However, due to its preservation in alcohol for twenty years, the sclerotised spot on the penis tube is no longer clearly visible (Fig. 4). During this process, we found that the description and illustrations of *K.hunanensis* (see fig. 8 in Li and Li (2022)) from Hunan Province are highly consistent with those of *K.magnimacula* (Figs 3, 4). Additionally, Guizhou Province borders Hunan Province and the type localities of these two species are relatively close, being only about 280 kilometres apart (Fig. 8). Therefore, we consider *K.hunanensis* as a synonym of *K.magnimacula* in this paper.

### Kamimuria extremispina

Du, 2006

60ADDAB2-3F74-5FAA-95E0-79393AEA1522

Kamimuria extremispina Du, 2006: 87. Stark & Sivec, 2013: 117. Yang & Li, 2018: 29.
Kamimuria circumspina Li, Mo & Yang, 2019: 139. syn. nov.
Kamimuria dabieshana Yan, Kong & Li, 2021: 550. syn. nov.

#### Materials

- **Type status:** Holotype. **Occurrence:** recordedBy: Sun Changhai; individualCount: 1; sex: male; lifeStage: adult; **Location:** country: China; stateProvince: Jiangxi; locality: 38 km north of Wuyishan National Nature Reserve (formerly known as Chong’an); **Event:** year: 1990; month: 5; day: 29
- **Type status:** Paratype. **Occurrence:** recordedBy: Du Yuzhou; individualCount: 3; sex: 2 males, 1 female; lifeStage: adult; **Location:** country: China; stateProvince: Guizhou; county: Jiangkou; locality: Fanjing Mountain, Heiwan River.; **Event:** year: 1994; month: 6; day: 19
- **Type status:** Other material. **Occurrence:** recordedBy: Xue Haiyang; individualCount: 5; sex: 3 males, 2 females; **Location:** country: China; stateProvince: Fujian; county: Nanping; locality: Mount Wuyi; **Event:** year: 2009; month: 6; day: 2

#### Distribution

China (Fujian, Guizhou, Guangxi, Hubei, Jiangxi)

#### Taxon discussion

**Figure 5.**
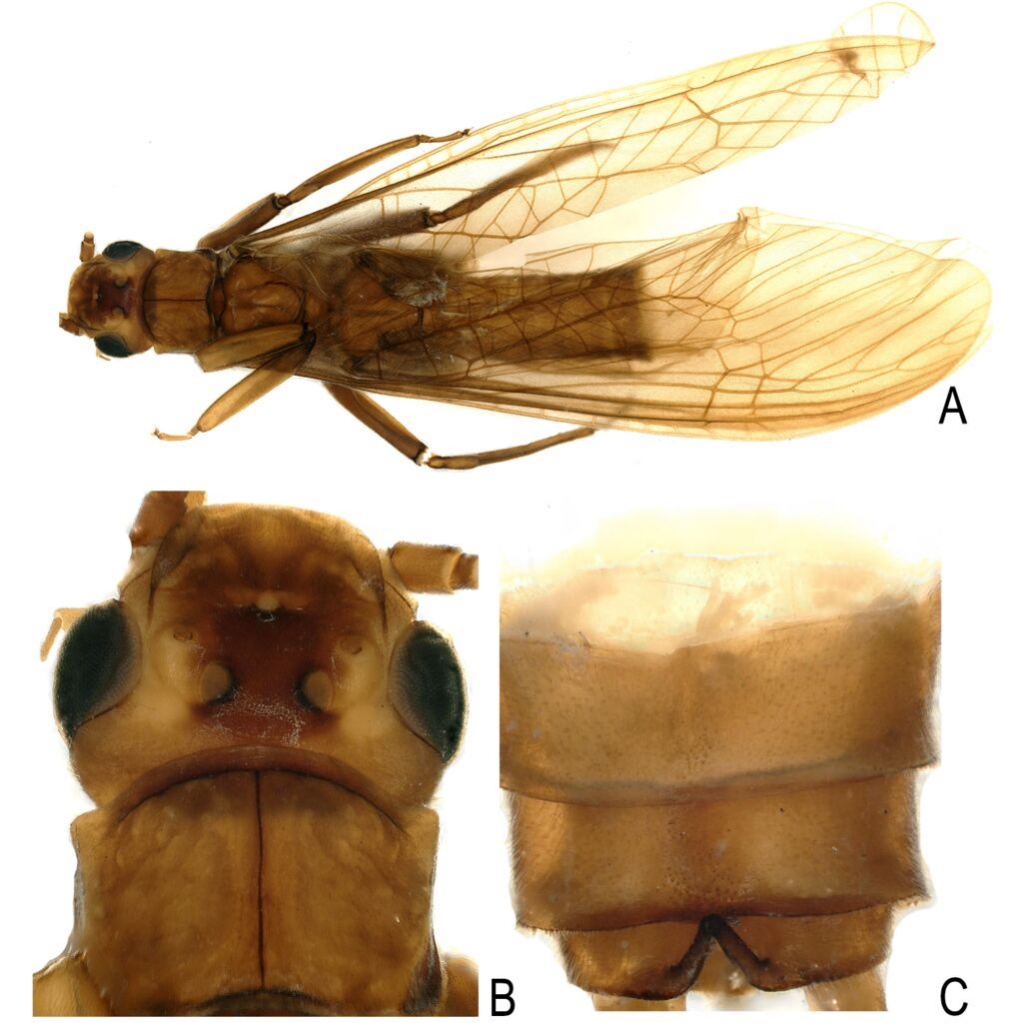
*Kamimuriaextremispina*, male, paratype. **A** dorsal habitus; **B** head and pronotum, dorsal view; **C** abdominal terminalia, dorsal view.

**Figure 6.**
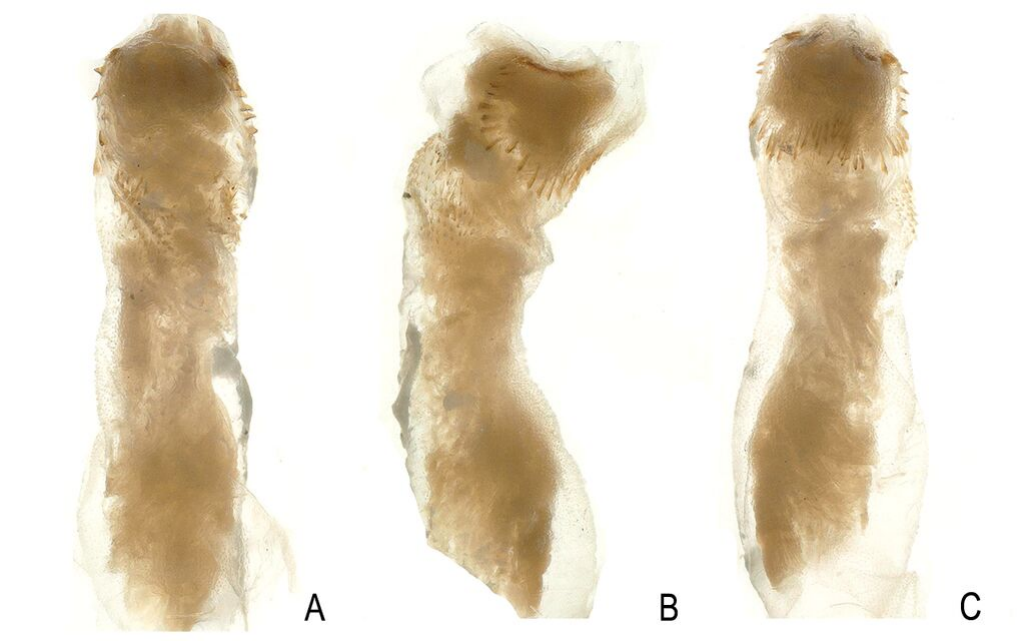
*Kamimuriaextremispina*, male holotype, everted penis. **A** dorsal view; **B** lateral view; **C** ventral view.

**Figure 7.**
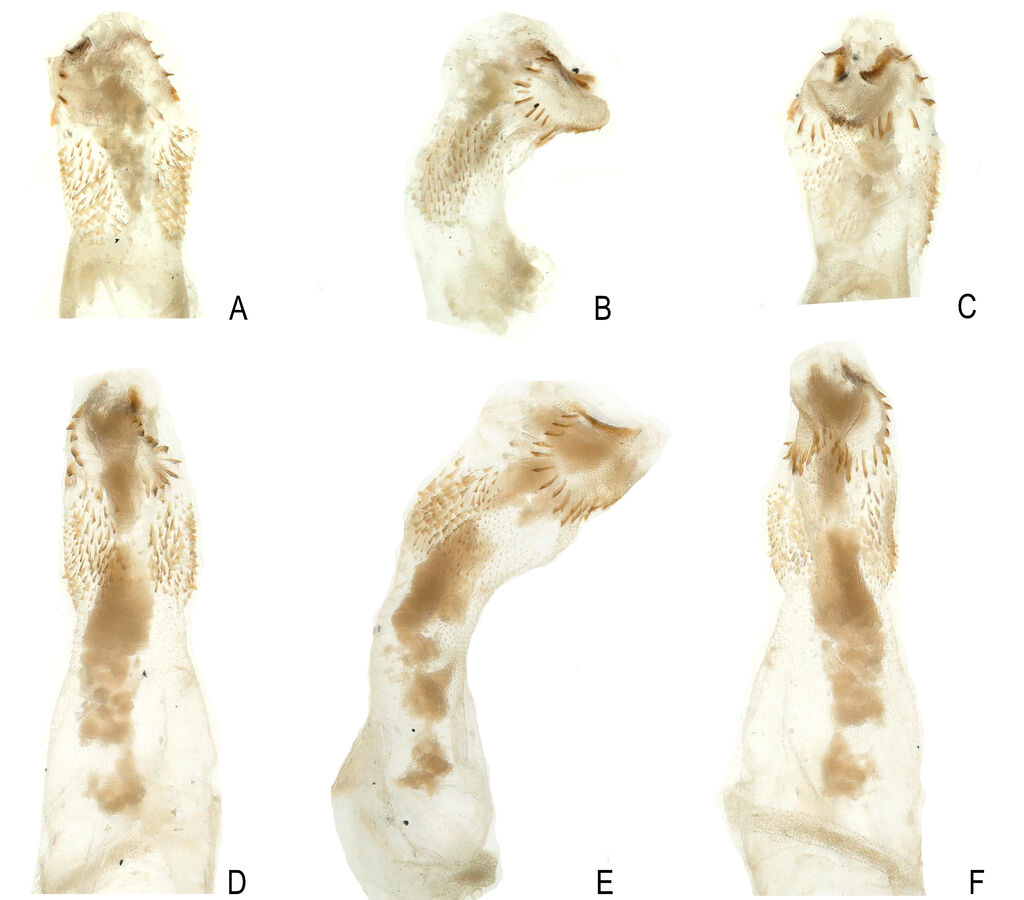
*Kamimuriaextremispina*. **A-C** Paratype, everted penis, dorsal view (A), lateral view (B) and ventral view (C); **D-F** Specimen from Fujian, dorsal view (D), lateral view (E) and ventral view (F).

**Figure 8.**
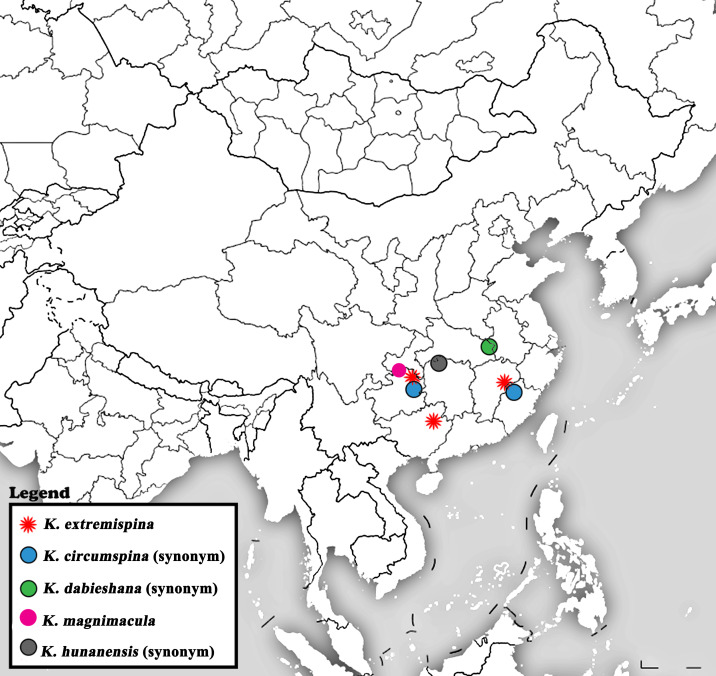
Distribution of *Kamimuriaextremispina* and *K.magnimacula*. (*K.extremispina* = *K.circumspina* syn. nov. = *K.dabieshana* syn. nov. and *K.magnimacula* = *K.hunanensis* syn. nov.).

The type locality of *Kamimuriacircumspina* is Foding Mountain in Guizhou Province, China, with additional distribution records from Mount Wuyi in Fujian Province (Yan et al. 2021). Huo et al. (2022b) provided supplementary descriptions of *K.simplex* (Chu, 1929), based on adult specimens collected from Mount Wuyi, Fujian Province. However, Kang et al. (2025) revised *K.simplex* and suggested that Huo’s specimens should be identified as *K.circumspina*. We examined the type specimens of *K.extremispina* and specimens of *K.circumspina* collected from Mount Wuyi in Fujian Province (see fig. 8 in Li et al. (2019)), finding that the male adult morphology and penis characteristics of the two species are highly consistent (Figs 5, 6, 7). Additionally, the holotype of *K.extremispina* was collected 38 km north of Mount Wuyi in Jiangxi Province (formerly known as Chong’an) and the paratype originates from Fanjing Mountain in Guizhou Province (Fig. 8), both of which are geographically close to the distribution range of *K.circumspina*. Therefore, we propose that *K.circumspina* should be regarded as a synonym of *K.extremispina*.

Another species, *K.dabieshana*, was described from Dabie Mountain in Hubei Province and was compared with its closely-related *K.circumspina*. The differences in the penis between the two species are as follows: in *K.dabieshana*, the apical two-thirds of the penis are distinctly constricted, with a small dorsal lobe preceding the constriction. The differences in male adults are as follows: the sensilla on tergum 9 of *K.dabieshana* are pale brown, whereas *K.circumspina* has black tergal sensilla, as well as differences in the curvature of the hemitergal lobes (Yan et al. 2021). However, *Kamimuria* species from different regions exhibit variable morphological characteristics, including thoracic and abdominal patterns, the number of basiconica sensilla and the shapes of the hemiterga (Huo et al. 2022b). Furthermore, intraspecific morphological variation in stoneflies, such as differences in head patterns, sensilla patches, wing venation and subgenital plates, has been well-documented in Chinese Perlodidae (Zwick 1997, Huo et al. 2020, Huo et al. 2022a). Given this established variability, we consider the observed differences between *K.dabieshana* and *K.circumspina* to represent normal intraspecific variation, potentially influenced by factors such as photographic angles, penis dissection techniques or specimen preservation. These variations are insufficient to justify the establishment of a new species. In conclusion, we propose that *K.dabieshana* should be regarded as a synonym of *K.extremispina*.

**Figure 9.**
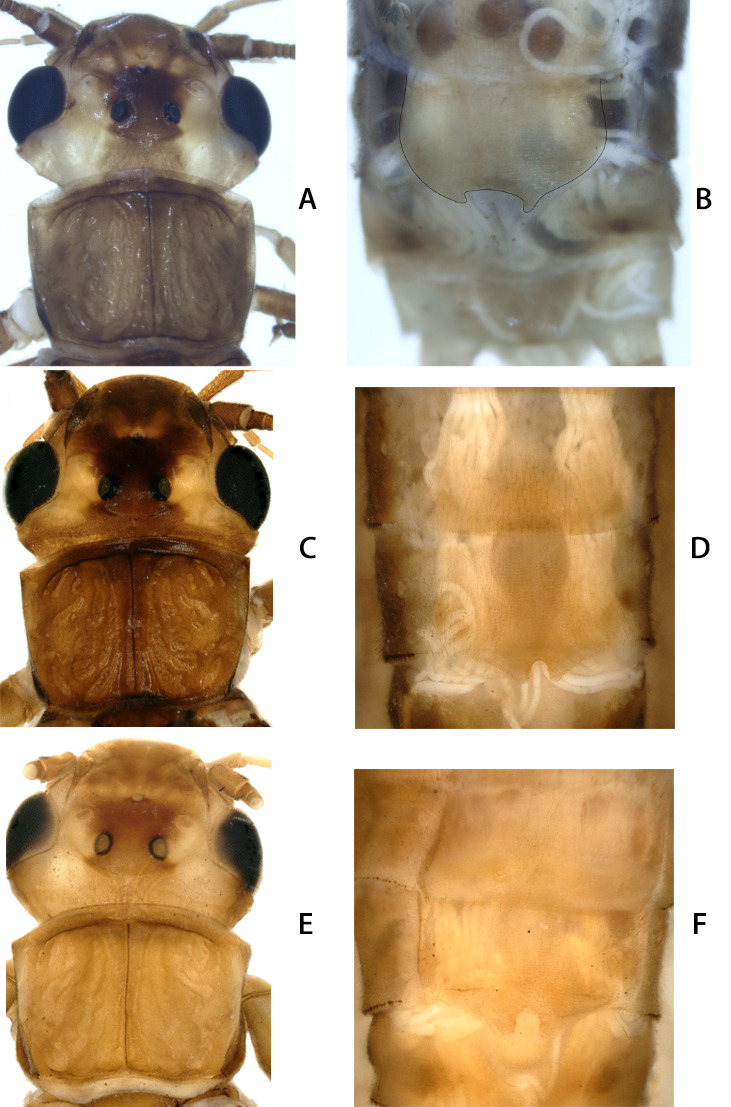
*Kamimuriaextremispina*, female. **A, C, E**: Head and pronotum, dorsal view; **B, D, F**: Terminalia, ventral view. **A–B** from Hubei (Yan et al. 2021); **C–D** from Fujian; **E–F** from Guizhou.

After a comparative morphological analysis of the male of *Kamimuriaextremispina*, *K.circumspina* and *K.dabieshana*, we concluded that *K.circumspina* and *K.dabieshana* should be treated as synonyms of *K.extremispina*. Considering that the morphological features of female specimens are less susceptible to variations caused by photographic angles, dissection techniques or preservation conditions, we conducted a morphological comparison of female specimens collected from Hubei, Fujia and Guizhou Provinces. Despite nearly 20 years of alcohol immersion causing the head patterns of the Guizhou specimens to fade, the markings are still consistent with those of the Hubei and Fujian specimens, exhibiting the following characteristics: black markings covering the ocellar area, with the patch extending anterolaterally between the M-line and the tentorial callosites and frons brownish to brown (Yan et al. 2021). Regarding the abdominal structure of female specimens, the subgenital plate of the female from Hubei is much wider, covering 2/3 width of the segment and the posteromedial notch between the posterior lobes is shallow, but wide. In comparison, the subgenital plate of the female from Fujian and Guizhou is narrower, approximately half the width of the segment and the posteromedial notch is deep and narrow arch-like (Fig. 9). The morphological variation in the subgenital plate exhibits a continuous and gradual transition amongst these specimens.

## Supplementary Material

XML Treatment for Kamimuria magnimacula

XML Treatment for Kamimuria extremispina
